# Newly diagnosed and previously treated multicentric Castleman disease respond equally to siltuximab

**DOI:** 10.1111/bjh.17177

**Published:** 2020-10-31

**Authors:** Frits van Rhee, Jean‐François Rossi, David Simpson, Alexander Fosså, Angela Dispenzieri, John Kuruvilla, Yeow Tee Goh, Seok‐Goo Cho, Marcelo Capra, Ting Liu, Corey Casper, James Cavet, Raymond S. Wong

**Affiliations:** ^1^ Myeloma Center University of Arkansas for Medical Sciences Little Rock AR USA; ^2^ Institut Sainte Catherine Avignon France; ^3^ BeiGene San Mateo CA USA; ^4^ Oslo University Hospital Oslo Norway; ^5^ KG Jebsen Centre for B cell malignancies Oslo Norway; ^6^ Mayo Clinic Rochester MN USA; ^7^ Princess Margaret Cancer Centre Toronto Canada; ^8^ Singapore General Hospital Singapore Singapore; ^9^ Seoul St. Mary's Hospital The Catholic University of Korea Seoul Republic of Korea; ^10^ Instituto do Cancer, Hospital Mãe de Deus Porto Alegre Brazil; ^11^ West China Hospital of Sichuan University Chengdu China; ^12^ Infectious Disease Research Institute Seattle WA USA; ^13^ Fred Hutchinson Cancer Research Center Seattle WA USA; ^14^ University of Washington Seattle WA USA; ^15^ Christie Hospital & University of Manchester Manchester UK; ^16^ Sir YK Pao Centre for Cancer Prince of Wales Hospital The Chinese University of Hong Kong Shatin Hong Kong

**Keywords:** siltuximab, multicentric Castleman disease

Multicentric Castleman disease (MCD) is a lymphoproliferative disorder characterised by systemic symptoms, such as fatigue, fever, night sweats and weight loss, as well as multistation lymphadenopathy. Laboratory abnormalities include anaemia, hypoalbuminaemia and elevated acute‐phase reactants, for example, C‐reactive protein (CRP) and erythrocyte sedimentation rate (ESR).^1^, ^2^, ^3^, ^4^ MCD is associated with human herpesvirus‐8 (HHV‐8) infection in immunocompromised patients. However, MCD is unrelated to HHV‐8 in up to 50% of patients^5^; this disease entity is known as HHV8‐negative or idiopathic MCD (iMCD). Interleukin (IL)‐6 plays a central role in the pathogenesis of iMCD, with multiple pro‐inflammatory effects.^6^, ^7^

Siltuximab is a monoclonal antibody that specifically binds human IL‐6 with high affinity and prevents it from interacting with the IL‐6 receptor complex, thereby inactivating IL‐6‐induced signalling.^8^, ^9^ In the Phase II, randomised, placebo‐controlled study in HIV‐ and HHV8‐negative patients with MCD, siltuximab plus best supportive care (BSC) led to a significant improvement in durable tumour and symptomatic response (34% vs. 0% with placebo plus BSC; *P* = 0·0012) (ClinicalTrials.gov identifier: NCT01024036).^10^ Primarily based on the results of this trial, siltuximab was approved by the United States Food and Drug Administration and the European Medicines Agency for the treatment of iMCD.^11^, ^12^ We report here a prespecified analysis of the efficacy and safety of siltuximab in this trial of patients who were either newly diagnosed or had received prior therapy.

Eligible patients had symptomatic, centrally confirmed HIV‐ and HHV8‐negative MCD. Prior IL‐6‐targeted therapy (e.g. tocilizumab) was not permitted. Patients receiving concomitant corticosteroids were considered for study inclusion provided the dose did not exceed 1 mg/kg/day of prednisone or equivalent and had remained stable or decreased over the preceding 4 weeks. Patients were randomly assigned in a 2:1 ratio to receive siltuximab (11 mg/kg) or placebo every 3 weeks until treatment failure. Randomisation was stratified by concomitant corticosteroid use. The primary and secondary efficacy endpoints, safety and statistical methods have been reported.^10^

In all, 79 patients were randomised: 53 were assigned to siltuximab and 26 to placebo. Of these, 46 patients were previously treated (siltuximab, *n* = 29; placebo, *n* = 17) and 33 were newly diagnosed (siltuximab, *n* = 24; placebo, *n* = 9) (Table SI). There were no significant differences in baseline characteristics, with the exception of histological subtype (*P = *0·031): the hyaline vascular subtype was more common among newly diagnosed patients, while more previously treated patients had mixed histology. Details of prior regimens are shown in Table SII. The median duration of treatment was 375 and 233 days in the siltuximab and placebo arms, respectively. Durable tumour and symptomatic response rates were similar for siltuximab‐treated patients compared with placebo in the previously treated (34·5% [10/29] vs. 0% [0/17]; *P* = 0·013) and newly diagnosed (33·3% [8/24] vs. 0% [0/9]; *P* = 0·09) subgroups (Table I). The median time to treatment failure (TTF) was not reached with siltuximab in both subgroups. For the placebo group, the median TTF was 184 days for previously treated patients (hazard ratio [HR] 0·60, 95% confidence interval [CI] 0·26–1·38; *P* = 0·23) and 106 days for newly diagnosed patients (HR 0·19, 95% CI 0·06–0·61; *P* = 0·005) (Fig 1, Figure S1). In patients treated with siltuximab, the median TTF appeared to be longer for newly diagnosed patients compared with previously treated patients; however, a Cox interaction analysis showed there was no significant difference between treatment effect and prior treatment status (*P* = 0·11). Results of the other secondary endpoints consistently favoured siltuximab over placebo in both the previously treated and newly diagnosed MCD subgroups, although statistical significance was not always reached (Table SIII). In the previously treated subgroup, the numbers of patients treated with a particular prior therapy (e.g. rituximab) were too small to draw meaningful conclusions on efficacy parameters after that particular treatment.

**Table I:** Durable tumour and symptom response. Prespecified subgroup analysis by treatment arm of the primary endpoint (durable tumour response by central review in the absence of symptom deterioration) in patients with previously treated or newly diagnosed multicentric Castleman disease.

	Newly diagnosed		Previously treated	
	Placebo (*n* = 9)	Siltuximab (*n* = 24)	Placebo (*n* = 17)	Siltuximab (*n* = 29)
Best overall response, *n* (%)				
Complete response	0 (0)	1 (4)	0 (0)	0 (0)
Partial response	0 (0)	7 (29)	0 (0)	10 (34)
Stable disease	7 (78)	16 (67)	15 (88)	15 (52)
Progressive disease	2 (22)	0 (0)	2 (12)	4 (14)
Overall response rate, *n* (%)	0 (0)	8 (33)	0 (0)	10 (34)
*P*	0·0891		0·0126	

**Fig 1:**
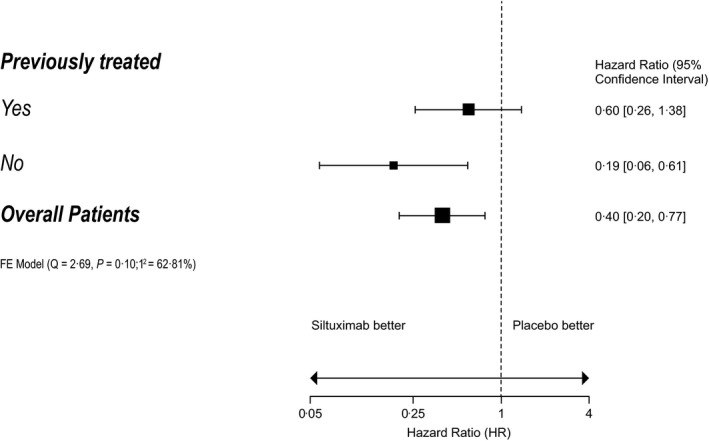
Hazard ratios (HRs) for benefit from siltuximab (time to treatment failure) in previously treated and newly diagnosed patients. A fixed effects (FE) model was used to compare HR results between the previously treated and untreated arms. Treatment failure defined as any of the following: increase from baseline in disease‐related Grade ≥2 symptoms for ≥3 weeks; any new disease‐related Grade ≥3 symptom; sustained (i.e. ≥3 weeks) increase from baseline in ECOG Performance Status by >1 point; radiological progression as measured by modified Cheson criteria; or initiation of any other MCD therapy. ECOG, Eastern Cooperative Oncology Group; MCD, multicentric Castleman disease.

The populations of the subgroups were relatively small, limiting the value of comparing adverse event (AE) frequencies; however, the general trends suggest that the frequencies of AEs, Grade ≥3 AEs and serious AEs were similar across the previously treated and newly diagnosed subgroups, despite longer treatment duration in the siltuximab arm (Table SIV).

This prespecified subgroup analysis showed that siltuximab plus BSC demonstrated efficacy in both previously treated and newly diagnosed patients, with durable symptomatic and tumour responses being achieved significantly more often with siltuximab than with placebo in both subgroups, and at similar rates (34·5% and 33·3%, respectively).

Secondary efficacy endpoints also consistently favoured siltuximab *versus* placebo in both subgroups, including TTF, tumour response rate, durable symptomatic response, reduction in serum CRP levels and ≥15 g/l increase in haemoglobin concentration (Figure S2). This suggests that there is no evidence of cross‐resistance with previously used agents, consistent with the novel mechanism of action of bioactive IL‐6 neutralisation.

The TTF appeared to be different when comparing the curves for newly diagnosed and previously treated patients, with the difference between siltuximab and placebo in previously treated patients not being significant, despite the separation of the curves. When looking at durable symptom responses in previously treated patients, the responses were higher with siltuximab than with placebo (45% vs. 24%, respectively). The failure of some of the outcomes in previously treated patients to reach statistical significance may have been caused by a number of factors, including the impact of prior therapies, small numbers in the respective subgroups and possibly variations in concomitant steroid use (31% siltuximab vs. 17% placebo).

Of the 53 patients overall who received siltuximab treatment, 31 were still on therapy at the end of the study, and 28 enrolled in the long‐term safety extension study.^12^ Patients enrolled in the extension study, which comprised patients from this study and those who participated in the Phase I study,^9^ were followed for a further 6 years on open‐label siltuximab, and 70% of those patients were still experiencing disease control.^12^

This prespecified analysis provides further support to the recommendation by the Castleman Disease Collaborative Network (CDCN) that siltuximab should be given as first‐line therapy to all patients with iMCD.^13^

## Conflicts of interest

Frits van Rhee reports receiving research support from Janssen Pharmaceuticals and consultant fees from EUSA Pharma. James Cavet reports receiving research funding, speaker fees and conference support from Janssen/Johnson & Johnson (J&J) and serving on an advisory board for EUSA Pharma. Angela Dispenzieri reports receiving research funding from Alnylam, Celgene, Pfizer and Takeda, and serving on advisory boards for Akcea, Intellia and Janssen. Jean‐François Rossi is the co‐founder of E‐Sana and reports having served on advisory boards for EUSA Pharma, LEO Pharma and Petrovax Pharm. John Kuruvilla reports having received honoraria and research funding from Janssen. Raymond S. Wong reports having received research funding from and served on an advisory board for Janssen/J&J, and speaker fees and conference support from EUSA Pharma and Janssen/J&J. The other authors have no conflicts of interest to disclose.

## Author contributions

Frits van Rhee contributed to the study design. Frits van Rhee, Jean‐François Rossi, David Simpson, Alexander Fosså, John Kuruvilla, Yeow Tee Goh, Seok‐Goo Cho, Marcelo Capra, Ting Liu, Corey Casper, James Cavet and Raymond S. Wong contributed to the data collection. Frits van Rhee and James Cavet analysed the data. Frits van Rhee, David Simpson, Alexander Fosså, Angela Dispenzieri, John Kuruvilla, Yeow Tee Goh, Seok‐Goo Cho, Marcelo Capra, Corey Casper and James Cavet interpreted the data, and all the authors provided critical review of the paper.

## Supporting information


**Fig S1**. The median time to treatment failure. Prespecified subgroup analysis in patients with newly diagnosed or previously treated multicentric Castleman disease. Treatment failure defined as any of the following: increase from baseline in disease‐related Grade ≥2 symptoms for ≥3 weeks; any new disease‐related Grade ≥3 symptom; sustained (i.e. ≥3 weeks) increase from baseline in ECOG Performance Status by >1 point; radiological progression as measured by modified Cheson criteria; or initiation of any other MCD therapy. TTF, time to treatment failure; d, days; HR, hazard ratio; ECOG, Eastern Cooperative Oncology Group; MCD, multicentric Castleman disease.

**Fig S2**. Serum C‐reactive protein. Median serum concentrations of C‐reactive protein at each treatment cycle in (A) newly diagnosed patients and (B) previously treated patients.

**Table SI**. Baseline patient demographics and disease characteristics.
**Table SII**. Prior treatment regimens in previously treated patients.
**Table SIII**. Secondary efficacy endpoints.
**Table SIV**. Number of subjects with treatment‐emergent adverse events of any grade (occurring in ≥10% of patients) or Grade ≥3 (occurring in ≥5% of patients).
