# Supplementary material for: Newly diagnosed and previously treated multicentric Castleman disease respond equally to siltuximab
Source: Br J Haematol. 2020 Oct 31;192(1):e28–31. doi: 10.1111/bjh.17177 (PMC7820993; doi:10.1111/bjh.17177)
Supplement: Supplementary file 1 — Fig S1. The median time to treatment failure. Prespecified subgroup analysis in patients with newly diagnosed or previously treated multicentric Castleman disease. Treatment failure defined as any of the following: increase from baseline in disease‐related Grade ≥2 symptoms for ≥3 weeks; any new disease‐related Grade ≥3 symptom; sustained (i.e. ≥3 weeks) increase from baseline in ECOG Performance Status by >1 point; radiological progression as measured by modified Cheson criteria; or initiation of any other MCD therapy. TTF, time to treatment failure; d, days; HR, hazard ratio; ECOG, Eastern Cooperative Oncology Group; MCD, multicentric Castleman disease. [file BJH-192-e28-s001.pdf]

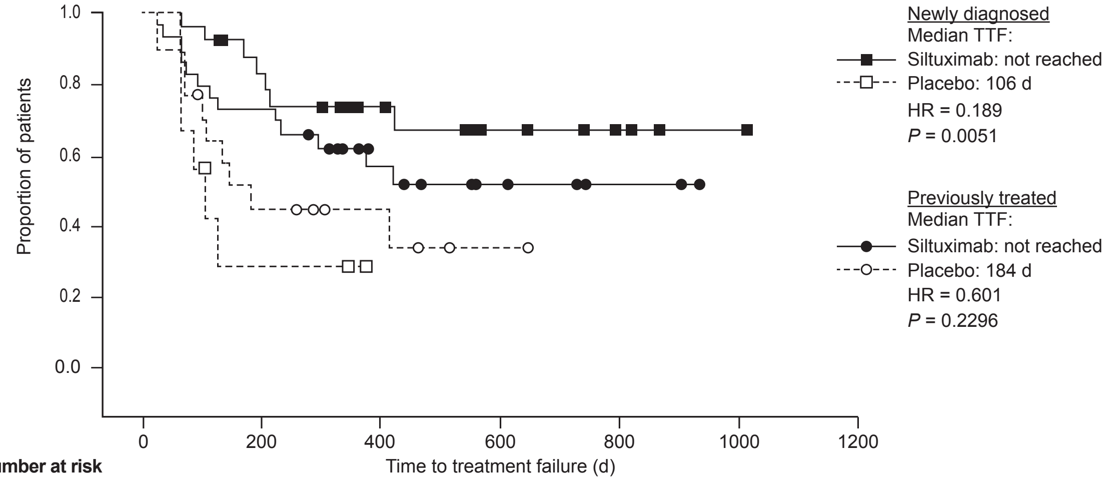

**Number at risk**							
Siltuximab – newly diagnosed	24	18	12	7	3	1	0
Placebo – newly diagnosed	9	2	0	0	0	0	0
Siltuximab – prior treatment	29	21	11	5	2	0	0
Placebo – prior treatment	17	7	4	1	0	0	0
