# Supplementary figures and images for: Newly diagnosed and previously treated multicentric Castleman disease respond equally to siltuximab
Source: Br J Haematol. 2020 Oct 31;192(1):e28–31. doi: 10.1111/bjh.17177 (PMC7820993; doi:10.1111/bjh.17177)

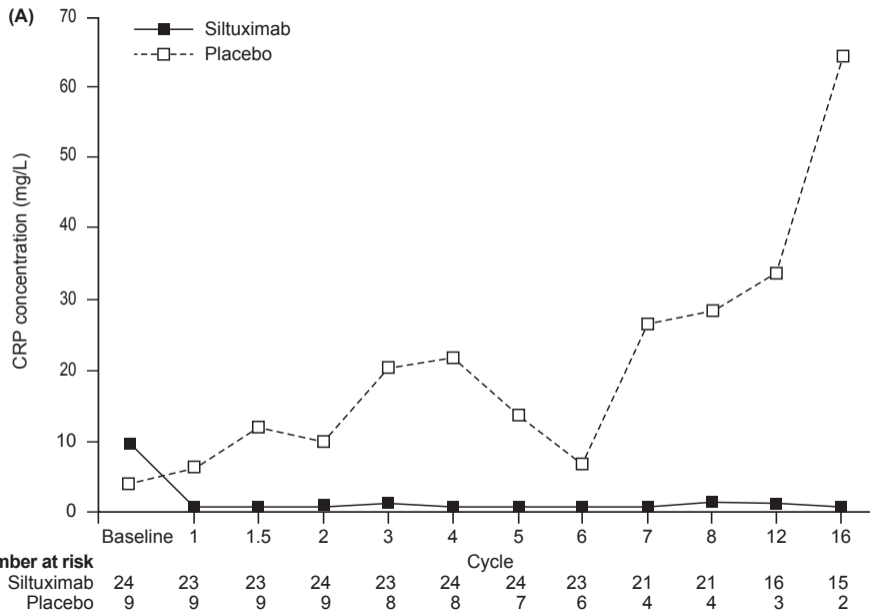

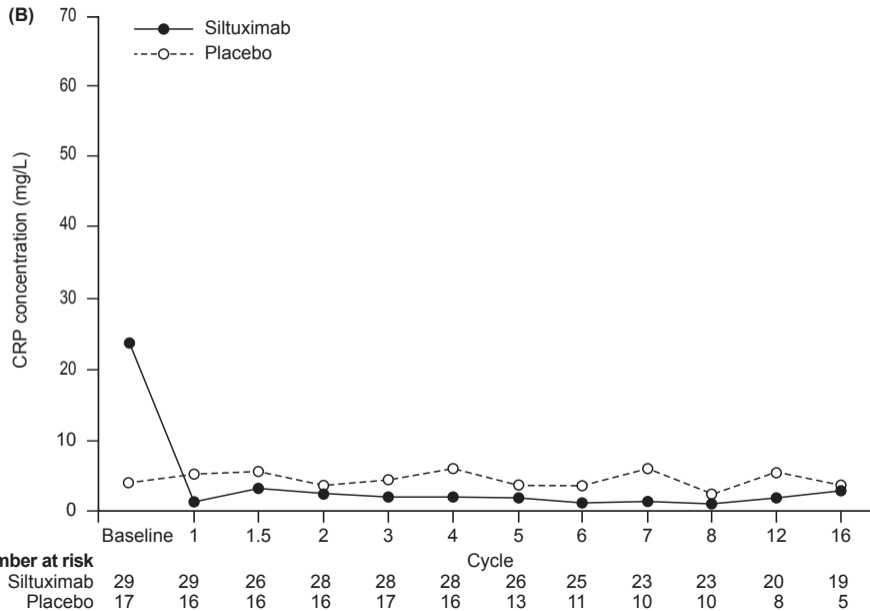

Supplement: Supplementary file 2 — Fig S2. Serum C‐reactive protein. Median serum concentrations of C‐reactive protein at each treatment cycle in (A) newly diagnosed patients and (B) previously treated patients. [file BJH-192-e28-s002.pdf]
