# Supplementary material for: Newly diagnosed and previously treated multicentric Castleman disease respond equally to siltuximab
Source: Br J Haematol. 2020 Oct 31;192(1):e28–31. doi: 10.1111/bjh.17177 (PMC7820993; doi:10.1111/bjh.17177)
Supplement: Supplementary file 3 — Table SI. Baseline patient demographics and disease characteristics. Table SII. Prior treatment regimens in previously treated patients. Table SIII. Secondary efficacy endpoints. Table SIV. Number of subjects with treatment‐emergent adverse events of any grade (occurring in ≥10% of patients) or Grade ≥3 (occurring in ≥5% of patients). [file BJH-192-e28-s003.docx]

**Supplementary Table I.** Baseline patient demographics and disease characteristics.

|  | Newly diagnosed | | Previously treated | |
| --- | --- | --- | --- | --- |
|  | Placebo  (*n* = 9) | Siltuximab  (*n* = 24) | Placebo  (*n* = 17) | Siltuximab  (*n* = 29) |
| Median age (range), years | 44 (27–78) | 43 (20–74) | 48 (29–62) | 48 (20–64) |
| Male sex, *n* (%) | 7 (78) | 13 (54) | 15 (88) | 17 (59) |
| Race, *n* (%)  White  Asian  Other | 6 (67)  2 (22)  1 (11) | 10 (42)  10 (42)  4 (17) | 6 (35)  9 (53)  2 (12) | 9 (31)  17 (59)  3 (10) |
| ECOG performance status, *n* (%)  0  1  2 | 4 (44)  5 (56)  0 (0) | 10 (42)  13 (54)  1 (4) | 6 (35)  11 (65)  0 (0) | 12 (41)  11 (38)  6 (21) |
| Histology, *n* (%)  Hyaline vascular  Plasmacytic  Mixed | 4 (44)  2 (22)  3 (33) | 11 (46)  7 (29)  6 (25) | 4 (24)  3 (18)  10 (59) | 7 (24)  6 (21)  16 (55) |
| Median disease-related overall symptom score (range) | 10 (4–16) | 5·5 (2–31) | 7 (1–30) | 6 (2–20) |
| Corticosteroid use at baseline,  *n* (%) | 2 (22) | 4 (17) | 7 (41) | 9 (31) |
| Median CRP (range), mg/L | 3·9  (0·8–81·2) | 9·8  (0·5–144·0) | 4·3  (0·4–107·0) | 23·7  (1·0–181·0) |
| Median haemoglobin (range), g/L | 130·3  (95–157) | 121·5  (81–147) | 134·0  (84–174) | 117·0  (70–175) |
| Median fibrinogen (range), μmol/L | 11·6  (7·3–25·8) | 12·1  (6·9– >29·4) | 12·1  (9·0– >29·4) | 13·9  (7·5–>29·4) |
| Median IgG (range), g/L | 12·6  (5·0–41·2) | 20·8  (6·4–67·8) | 17·6  (4·7–68·2) | 15·2  (5·9–108·0) |

ECOG, Eastern Cooperative Oncology Group; CRP, C-reactive protein; IgG, immunoglobulin G.

**Supplementary Table II.** Prior treatment regimens in previously treated patients.

| All patients | Placebo  (*n* = 17) | Siltuximab  (*n* = 29) | Combined  (*N* = 46) |
| --- | --- | --- | --- |
| No. of prior regimens, *n* (%)  1  2  3  >3 | 8 (47%)  4 (24%)  1 (6%)  4 (24%) | 18 (62%)  4 (14%)  4 (14%)  3 (10%) | 26 (57%)  8 (17%)  5 (11%)  7 (15%) |
| Components of regimen,* *n* (%)  Corticosteroids  Cyclophosphamide  Vincristine  Rituximab  Doxorubicin  Vindesine  Etoposide  Thalidomide | 15 (88%)  8 (47%)  5 (29%)  3 (18%)  1 (6%)  0  0  2 (12%) | 28 (97%)  15 (52%)  7 (24%)  5 (17%)  4 (14%)  4 (14%)  3 (10%)  1 (3%) | 43 (93%)  23 (50%)  12 (26%)  8 (17%)  5 (11%)  4 (9%)  3 (7%)  3 (7%) |

*In ≥5% previously treated patients; those treated with tocilizumab were excluded from the study.

**Supplementary** **Table III.** Secondary efficacy endpoints.

| Endpoint | Placebo | Siltuximab | *P*-value |
| --- | --- | --- | --- |
| *n*, (%) |  |  |  |
| Durable tumour and symptomatic response by investigator assessment*  All patients  Newly diagnosed  Previously treated | 0 (0%)  0 (0%)  0 (0%) | 24 (45%)  10 (42%)  14 (48%) | <0·0001  0·0368  0·0013 |
| Tumour response by independent assessment  All patients  Newly diagnosed  Previously treated | 1 (4%)  1 (11%)  0 (0%) | 20 (38%)  10 (42%)  10 (34%) | 0·0051  0·1941  0·0208 |
| Tumour response by investigator assessment  All patients  Newly diagnosed  Previously treated | 0 (0%)  0 (0%)  0 (0%) | 27 (51%)  11 (46%)  16 (55%) | <0·0001  0·0224  0·0003 |
| Durable symptomatic response  All patients  Newly diagnosed  Previously treated | 5 (19%)  1 (11%)  4 (24%) | 30 (57%)  17 (71%)  13 (45%) | 0·0019  0·0040  0·1478 |
| Durable complete symptomatic response  All patients  Newly diagnosed  Previously treated | 0 (0%)  0 (0%)  0 (0%) | 13 (25%)  8 (33%)  5 (17%) | 0·0103  0·0891  0·1290 |
| Haemoglobin response ≥1.5 g/dL increase^†^  All patients  Newly diagnosed  Previously treated | 0 (0%)  0 (0%)  0 (0%) | 19 (61%)  9 (64%)  10 (59%) | 0·0007  0·0373  0·0160 |

Total study cohort: placebo, *n* = 26, siltuximab, *n* = 53; newly diagnosed: placebo, *n* = 9, siltuximab, *n* = 24; previously treated: placebo, *n* = 17, siltuximab, *n* = 29.

*Defined as a complete or partial response of enlarged lymph nodes or other measurable lesions by modified Cheson criteria, with improvement or stabilisation of disease-related symptoms for at least 18 weeks during masked treatment.

†Hb-evaluable cohort: placebo, *n* = 11, siltuximab, *n* = 31; newly diagnosed: placebo, *n* = 4, siltuximab, *n* = 14; previously treated: placebo, *n* = 7, siltuximab, *n* = 17.

*P*-values estimated using exact Cochrane–Mantel–Haentzel method, corrected for stratification factor (baseline corticosteroid use).

**Supplementary Table IV.** Number of subjects with treatment-emergent adverse events of any grade (occurring in ≥10% of patients) or Grade ≥3 (occurring in ≥5% of patients).

|  | Newly diagnosed | | | | Previously treated | | | |
| --- | --- | --- | --- | --- | --- | --- | --- | --- |
|  | Placebo (*n* = 9) | | Siltuximab (*n* = 24) | | Placebo  (*n* = 17) | | Siltuximab (*n* = 29) | |
| *n* (%) | All grades | Grade ≥3 | All grades | Grade ≥3 | All grades | Grade ≥3 | All grades | Grade ≥3 |
| **Generalised disorders and administration site conditions** | | | | | | | | |
| Oedema | 4 (44) | 1 (11) | 11 (46) | 2 (8) | 5 (29) | 1 (6) | 8 (28) | 2 (7) |
| Fatigue | 2 (22) | 0 (0) | 10 (42) | 3 (13) | 8 (47) | 1 (6) | 8 (28) | 2 (7) |
| Malaise | 2 (22) | 0 (0) | 7 (29) | 0 (0) | 3 (18) | 0 (0) | 8 (28) | 0 (0) |
| Weight decrease | 2 (22) | 0 (0) | 2 (8) | 0 (0) | 2 (12) | 0 (0) | 2 (7) | 0 (0) |
| Weight increase | 0 (0) | 0 (0) | 5 (21) | 1 (4) | 0 (0) | 0 (0) | 6 (21) | 1 (3) |
| Pyrexia | 0 (0) | 0 (0) | 2 (8) | 0 (0) | 2 (12) | 0 (0) | 4 (14) | 0 (0) |
| **Skin and subcutaneous tissue disorders** | | | | | | | | |
| Rash | 1 (11) | 0 (0) | 14 (58) | 0 (0) | 3 (18) | 0 (0) | 9 (31) | 1 (3) |
| Night sweats | 2 (22) | 0 (0) | 5 (21) | 2 (8) | 1 (6) | 1 (6) | 4 (14) | 2 (7) |
| Hyperhidrosis | 1 (11) | 0 (0) | 5 (21) | 2 (8) | 3 (18) | 0 (0) | 5 (17) | 0 (0) |
| Pruritus | 1 (11) | 0 (0) | 9 (38) | 0 (0) | 2 (12) | 0 (0) | 13 (45) | 0 (0) |
| **Infections and infestations** | | | | | | | | |
| Nasopharyngitis | 0 (0) | 0 (0) | 5 (21) | 0 (0) | 1 (6) | 0 (0) | 3 (10) | 0 (0) |
| Upper respiratory tract infection | 1 (11) | 0 (0) | 6 (25) | 0 (0) | 3 (18) | 1 (6) | 13 (45) | 0 (0) |
| **Respiratory, thoracic and mediastinal disorders** | | | | | | | | |
| Dyspnoea | 4 (44) | 1 (11) | 6 (25) | 0 (0) | 5 (29) | 0 (0) | 7 (24) | 1 (3) |
| Cough | 0 (0) | 0 (0) | 3 (13) | 0 (0) | 6 (35) | 0 (0) | 5 (17) | 0 (0) |
| **Metabolism and nutrition disorders** | | | | | | | | |
| Decreased appetite | 2 (22) | 0 (0) | 2 (8) | 1 (4) | 2 (12) | 0 (0) | 7 (24) | 0 (0) |
| Hypokalaemia | 0 (0) | 0 (0) | 2 (8) | 1 (4) | 2 (12) | 1 (6) | 4 (14) | 0 (0) |
| **Gastrointestinal disorders** | | | | | | | | |
| Nausea | 2 (22) | 0 (0) | 2 (8) | 1 (4) | 3 (18) | 0 (0) | 3 (10) | 0 (0) |
| Vomiting | 1 (11) | 0 (0) | 3 (13) | 1 (4) | 1 (6) | 0 (0) | 3 (10) | 0 (0) |
| Abdominal pain | 2 (22) | 1 (11) | 10 (42) | 0 (0) | 2 (12) | 1 (6) | 3 (10) | 0 (0) |
| Diarrhoea | 1 (11) | 0 (0) | 4 (17) | 0 (0) | 4 (24) | 1 (6) | 8 (28) | 0 (0) |
| **Blood and lymphatic system disorders** | | | | | | | | |
| Anaemia | 0 (0) | 0 (0) | 2 (8) | 0 (0) | 4 (24) | 3 (18) | 3 (10) | 1 (3) |
| Neutropenia | 0 (0) | 0 (0) | 4 (17) | 2 (8) | 2 (12) | 1 (6) | 3 (10) | 0 (0) |
| Thrombocytopenia | 1 (11) | 1 (11) | 5 (21) | 1 (4) | 0 (0) | 0 (0) | 3 (10) | 1 (3) |
| **Nervous system disorders** | | | | | | | | |
| Peripheral sensory neuropathy | 4 (44) | 1 (11) | 8 (33) | 0 (0) | 1 (6) | 0 (0) | 5 (17) | 0 (0) |
| Peripheral motor neuropathy | 0 (0) | 0 (0) | 2 (8) | 0 (0) | 2 (12) | 0 (0) | 4 (14) | 0 (0) |
| Dizziness | 0 (0) | 0 (0) | 3 (13) | 0 (0) | 2 (12) | 0 (0) | 3 (10) | 1 (3) |
| **Neoplasms benign, malignant and unspecified (including cysts and polyps)** | | | | | | | | |
| Tumour pain | 3 (33) | 0 (0) | 3 (13) | 0 (0) | 1 (6) | 0 (0) | 1 (3) | 0 (0) |
